# Hospital Subscriptions and Medical Schools

**Published:** 1905-02-25

**Authors:** 


### The Hospital.

A Journal of

The Medical Sciences and Hospital Administration.

Vol. XXXVII.—No. 961. FEBRUARY 25, 1905.

### HOSPITAL SUBSCRIPTIONS AND MEDICAL SCHOOLS.

A copy of the report of Sir Edward Fry's Committee appointed by King Edward's Hospital Fund, with the evidence and appendices, should be obtained and preserved by all who are really interested in the proper administration of a voluntary hospital. It must nip in the bud the tendency, which was growing up in the case of a few hospitals, to subscribe money from the general funds given for the relief of the sick poor, for the purposes of medical education. But, apart from this, in the report and evidence will be found many material facts which will shed a new light in many minds upon several branches of hospital administration which are occupying the thoughts of the public at the moment. The evidence, too, is especially interesting to all who know or have worked with the various gentlemen who gave evidence, for much of the latter is extremely characteristic. All who are interested in the voluntary hospitals are greatly indebted to the Prince of Wales and the King's Fund, and especially to Sir Edward Fry, the Bishop of Stepney, and Lord Welby, with Mr. Power their hon. secretary, for the large amount of time which they have so generously devoted to a somewhat intricate subject.

The whole of the report will be found on another page of the present issue. Briefly stated, its findings amount to this. In the case of King's College, Guy's, the Royal Free and University College Hospitals, their medical schools during 1903 derived no pecuniary benefit from the hospitals. On the other hand, contributions were directly or indirectly made in the same year to the medical schools out of th[illegible] funds of the London, Middlesex, St. Bartholom[illegible]w's, St. George's, St. Mary's, St. Thomas's, and th[illegible] Westminster Hospitals. In the case of each of these hospitals the schools remain debtors to the hospitals in respect of these pecuniary contributions, and no special considerations have been advanced in justification of such expenditure by the hospitals and the schools.

The evidence satisfies the commissioners, that the expenses incurred in the hospitals with schools are generally in excess of those at hospitals without schools, and they are of opinion that no saving in expense can be attributed to the presence of medical students. Indeed, the commissioners go further, for they point to remarkable variations in the expenses incurred by several hospitals to which schools are attached, where they appear to think that considerable extravagance and waste in expenditure may exist. A further finding is, that the material benefits received by the schools from the hospitals, and *vice versa*, may fairly be set off one against the other. Finally, the commissioners dispose of the whole question by recommending that in future the distinction between the hospital and the medical school shall in every case be drawn so that no question may arise as to the distinction and application of money contributed for the relief of the sick poor or otherwise. We welcome this report because it will relieve the hospitals from all risk that any money given to a hospital for one purpose will henceforward be applied to another. If the voluntary system of hospital support is to continue, it is essential that every penny subscribed shall be carefully safeguarded as to its expenditure, so that all contributors may have confidence, that business methods are enforced in regard to income, expenditure and relief.

In gifts to income there is a fixed, definite, maximum sum beyond which the English people cannot be expected to go. Nothing could prove more fatal to the revenue of a voluntary hospital than a feeling in the minds of the public that moneys entrusted to its managers were not economically and loyally expended upon the objects for which they were given. We need at the moment a revival in wise hospital economy, which will stimulate effort in cutting down expenditure wherever possible, and resistance to claims for all kinds of innovations due largely to competition, until the fullest consideration and the clearest evidence is forthcoming of the results which are likely to follow. Then too the amount and the quality of the relief afforded, and the social position of the patients, together with a clear recognition of the relative claims in proper order of each type of applicant is called for at the present time. The managers must prevent the use of beds, provided for the sick and needy residents of the metropolis, from being appropriated by non-residents from the country, who ought properly, for the most part at any rate, to receive their hospital benefits in institutions situated within the district where they reside. All these points are directly or indirectly raised by Sir Edward Fry's report, and no one who will take the trouble to read the evidence can fail to be interested or instructed. For these reasons, we regard the report as calculated to prove epoch-making and important.

